# Body appreciation and appearance evaluation in individuals with obesity compared to individuals with normal-weight: findings from a representative German population sample

**DOI:** 10.1007/s40519-020-01071-7

**Published:** 2020-12-05

**Authors:** Natascha-Alexandra Weinberger, Claudia Luck-Sikorski

**Affiliations:** 1 University of Applied Health Sciences SRH Gera, Neue Straße 28-30, 07548 Gera, Germany; 2 Integrated Research and Treatment Center (IFB) Adiposity Diseases, University Hospital Leipzig, Philipp-Rosenthal-Str. 27, 04103 Leipzig, Germany; 3 The Helmholtz Institute for Metabolic, Obesity and Vascular Research (HI-MAG), Helmholtz Zentrum München German Research Center for Environmental Health, Philipp-Rosenthal-Str. 27, 04103 Leipzig, Germany

**Keywords:** Body appreciation, Body image, Appearance evaluation, Obesity, Adults

## Abstract

**Purpose:**

Body image has been identified as an important factor in mental health in individuals with obesity. Previous studies have focused on negative body image and research of positive body image—particularly in obesity—is still in its infancy. The current study explored the positive body image facet body appreciation and the negative facet appearance evaluation in different BMI groups as well as men and women.

**Methods:**

A total of 1003 participants of the general German public above the age of 18 years completed measures on body appreciation and appearance evaluation.

**Results:**

Significantly lower body appreciation was observed in male participants with obesity compared to normal-weight participants. In women, the BMI groups did not differ significantly in body appreciation. BMI was negatively associated with appearance evaluation in both genders. While men and women did not differ in body appreciation, men reported lower appearance evaluation scores compared to women.

**Conclusion:**

The present study is one of few that investigated positive body image in individuals with obesity compared to individuals with normal weight. The findings underscore the potential of body appreciation as a resource in treatment of body image concerns in individuals with obesity. Further implications for future research are discussed.

**Level of evidence:**

III, case–control analytic study.

## Introduction

Due to its rising prevalence and thus significant contribution to the burden of disease, obesity has been dubbed a “global pandemic” [1]. Individuals with obesity are confronted with numerous physical as well psychosocial consequences [2, 3]. Among the latter, stigmatization and mental illness are of particular concern: Wu and Berry [4] report a positive link between weight stigma and eating disturbances, depression, anxiety and body dissatisfaction in adults with overweight and obesity. Further, a significant association between obesity and psychiatric conditions, particularly depression, has been found [5]. However, the relationship between the conditions is complex and numerous mediating factors have been subject of investigations for example with regard to prevention and treatment efforts [6]. As such, body image has been identified as an important factor in both, stigmatization and mental health in individuals with obesity [7].

The multidimensional construct of body image comprises cognitive, affective, behavioural, and perceptual aspects [8]. Particularly dissatisfaction with one’s body has been identified as predictor in the development of eating disorders [9]. While body image disturbance has frequently been studied in individuals with anorexia nervosa and bulimia nervosa [10], by now it has also gained significance in the research of overweight and obesity [11]. While previous studies have found individuals with obesity to report higher body dissatisfaction than normal-weight individuals [12], the relationship between body image and BMI is complex, and not all individuals with obesity report negative body image [13]: Individuals with obesity who are not seeking treatment for example have been found to report less body image concerns in comparison to those who are [14]. In line with this, for a considerable number of patients with obesity, body dissatisfaction is an even more important motivator to undergo bariatric surgery than health concerns [15]. Negative body image has also been identified as an important mediator in the relationship between BMI and mental illnesses like depression [16, 17].

In recent years the focus on the negative facets of body image has been called into question and prompted efforts to also understand and investigate positive body image [18]. Positive body image is a multifaceted construct and distinct from negative body image. One of its dimensions, body appreciation (BA), is defined as respecting and appreciating not just the body’s features, but also its function and health. Individuals with high BA take care of and accept their body and its unique features, view their body favorably, and do not agree with widely propagated appearance ideals [18, 19].

Previous research on BA reports positive associations with for example self-esteem and life satisfaction [20]. Moreover, health behavior such as regular physical activity and seeking medical attention have been found to be related to higher body appreciation levels [21, 22]. As such, BA is considered a potential protective factor for both physical and mental health [18]. Studies investigating BA in different BMI groups report an inverse relationship between BMI and BA for different ethnic groups [23]: Razmus [24] for example found lower levels of body appreciation in overweight/obese individuals compared to normal-weight individuals. However, there are also studies reporting mixed findings [25] or no relationship [26] between BMI and BA.

While efforts have been made in recent years to further research on body image in populations other than young, normal-weight women, there is still a lack of studies on the subject investigating men or individuals of different weight groups like overweight or obesity. Due to its relative novelty, this is even more relevant in research on positive body image. In one of few randomized prevention trials, Olson et al. [27] delivered a 4-week cognitive-dissonance-based body acceptance intervention (“The Body Project”) in combination with key components of weight loss treatment to women with overweight and obesity. The intervention was designed to help resist sociocultural pressures to conform to the thin-ideal and reduce the pursuit of thinness. The authors report the most considerable effects of their intervention for the body image facet BA, underlining its potential as a resource in treatment of body image concerns in obesity.

The current study aims to contribute to the investigation of positive body image, in particular BA in individuals with obesity in comparison to normal-weight individuals in Germany. To explore BA in the German public and to compare BA between different BMI groups as well as men and women, a representative sample was analyzed. In addition, appearance evaluation (AE) was included as negative body image facet to investigate differences as well as the relationship of the two body image facets with frequently correlated variables like gender and age. Based on previous research we hypothesized (a) women to report lower BA and AE scores compared to men and (b) individuals with obesity to report lower BA and AE scores compared to normal-weight individuals.

## Methods

### Participants

The total sample consisted of 450 men (48.9%) and 518 women (51.1%) of the general German public above the age of 18 years. An independent institute specializing in market, opinion, and social research (USUMA, Berlin, Germany) assisted in the drawing of a German population sample in May and June 2019. A three-stage random sampling procedure selected sample point regions based on representative data, target households within regions by means of a random route procedure, and target persons within households according to a kish selection grid. Following this procedure, 2335 noninstitutionalized civilians ≥ 18 years were randomly selected, and 1005 individuals participated in the survey, corresponding to a response rate of 43.0% (households: 375 not reached, 261 refused; target persons: 65 cancelled, 612 refused). Because of missing data, two participants were excluded, leaving a final sample of *N* = 1003 individuals. All participants gave verbal informed consent. The current study was approved by the local ethics committee (approval number: 2019-1279-Bef). Descriptive statistics of the sample are illustrated in Table 1.

**Table 1:** Descriptive statistics (mean and standard deviation) of sociodemographic data, depressive symptoms, self-esteem, body appreciation and appearance evaluation

	Total (*N* = 1003) *M* (SD)	Men (*n* = 490) *M* (SD)	Women (*n* = 513) *M* (SD)	Difference
Age	50.99 (18.98)	49.76 (18.49)	52.16 (19.39)	*p* = 0.046
Education^a^	2.98 (0.84)	2.92 (0.85)	3.05 (0.83)	*p* = 0.086
*n* (%) no degree	8 (0.8)	3 (0.7)	5 (1.0)	
*n* (%) lower secondary school	321 (33.7)	180 (38.3)	141 (29.3)	
*n* (%) secondary school	302 (31.7)	139 (29.5)	163 (33.9)	
*n* (%) high-school	322 (33.8)	148 (31.6)	173 (35.9)	
BMI^b^	25.98 (5.22)	26.97 (5.33)	25.00 (4.91)	*p* < 0.001
*n* (%) underweight	30 (3.0)	9 (1.9)	21 (4.0)	
*n* (%) normal-weight	426 (42.4)	166 (33.9)	260 (50.6)	
*n* (%) overweight	357 (35.6)	201 (41.1)	155 (30.3)	
*n* (%) obese	191 (19.0)	114 (23.2)	77 (15.1)	
Depressive symptoms^c^	4.89 (4.72)	4.83 (4.72)	4.94 (4.73)	*p* = 0.713
*n* (%) no depression	569 (57.8)	277 (58.7)	292 (57.1)	
*n* (%) mild	274 (27.8)	125 (26.5)	148 (28.9)	
*n* (%) moderate	94 (9.6)	48 (10.1)	47 (9.1)	
*n* (%) mod.-sev	29 (3.0)	14 (3.0)	15 (2.9)	
*n* (%) severe	18 (1.9)	8 (1.8)	10 (1.9)	
Self-esteem^d^	3.62 (0.94)	3.68 (0.94)	3.57 (0.93)	*p* = 0.060
BA	3.94 (0.61)	3.95 (0.59)	3.93 (0.62)	*p* = 0.497
AE	3.74 (0.69)	3.69 (0.68)	3.80 (0.69)	*p* = 0.013

^a^Participants’ educational attainment ranging from 1 = no school degree to 4 high-school degree

^b^BMI classes according to WHO [32]

^c^Higher PHQ-9 sum scores indicate higher severity of depressive symptoms: 5 and less = no depression, 5–10 = mild, 10–15 = moderate, 15–20 = moderate to severe, 20 and more = severe levels of depressive symptoms

^d^Participants’ self-reported self-esteem ranging from 1 = very low to 5 = very high

### Instruments

### Main outcomes

### Body appreciation scale (BAS)

The body appreciation scale was used as a measure of positive body image [20]. All 13 items are rated on a 5-point Likert-scale (1 = never to 5 = always). Scores are averaged to obtain an overall body appreciation (BA) score, with higher scores reflecting greater body appreciation. Swami and colleagues [25] report good internal reliability and construct validity for the scale’s German version. In the current sample, internal consistency was high both in men (*α* = 0.80) and women (*α* = 0.83).

### Multidimensional body-self relations questionnaire—appearance scales (MBSRQ—AS)

The German version of the Multidimensional Body-Self Relations Questionnaire—Appearance Scales (MBSRQ-AS; [28]) is a well-validated instrument [29] and consists of 34-items distributed among five subscales, which measure specific facets of appearance-related body image. The current study used the 7-item ‘Appearance Evaluation’ (AE) subscale of the MBSRQ-AS. It was rated on a 5-point Likert scale (1 = definitely disagree to 5 = definitely agree) and measures a participant’s general feelings of dissatisfaction with their physical appearance. Scores on each item were averaged to create a mean score, with lower scores indicating greater dissatisfaction with appearance. Overall internal consistency in the current sample was acceptable (*α* = 0.74).

### Correlates

Participants were asked to report their body height and weight as well as basic sociodemographic information (age, gender, educational attainment) at the beginning of the survey.

### The patient health questionnaire—depression module (PHQ-9)

Depressive symptoms were assessed using the depression module of the Patient Health Questionnaire (PHQ-9; [30]). Participants reported for each of nine depressive symptoms (based on DSM-IV criteria) whether the symptom had bothered them in the previous two weeks. The continuous variable was the sum of scores of the PHQ-9 items which ranges between 0 and 27. It was used to assess participants’ self-reported severity of depressive symptoms, with scores above 5, 10, 15 or more on the scale representing mild, moderate and moderately severe levels of depressive symptoms. Previous studies found the PHQ-9 to be a reliable and valid measure of depression severity [30]. Internal consistency was high in the current sample (*α* = 0.84).

### Self-esteem

Participants’ self-esteem was assessed using a single item rated on a 5-point Likert scale (1 = “I have very low self-esteem” to 5 = “I have very high self-esteem”).

### Statistical analyses

Data were analysed with SPSS 25.0.0 and weighted regarding to age, gender and education of the general population of Germany to control for bias. This variable was constructed by the research institute collecting the data using iterative proportional fitting procedure [31]. All analyses were run with and without the weight variable.

*T* tests and Mann–Whitney *U* tests were used to analyse differences between men and women in all variables of interest (see Table 1). Comparisons between BMI groups were analysed separately for men and women by one-way analysis of variance (Welch-ANOVA) with Games–Howell post-hoc tests for pair wise comparisons. Due to the low number of participants in the underweight group, normal-weight and underweight was collapsed into one category and analyses were run with four and three BMI groups respectively to ascertain potential differences. Multiple regression analyses were conducted to examine whether BA and AE respectively can be predicted based on BMI, gender, age, education, depressive symptoms, and self-esteem. Interaction terms were calculated using centred predictor variables to account for multicollinearity. *p* values were obtained from two-tailed tests, with a *p* < 0.05 indicating statistical significance.

## Results

Descriptive statistics of all variables of interest for the total sample as well as men and women separately are illustrated in Table 1. Reporting of weighted data is not done, as no differences were observed. Results for the analyses with the collapsed underweight/normal-weight category are reported, since analyses with four separate BMI groups did not yield different findings. Overall, female participants were significantly older, had a significantly lower BMI and self-esteem and reported significantly higher AE in comparison to male participants (see Table 1).

While no statistically significant difference was observed in women (Welch’s *F*(2, 192.22) = 1.84, *p* = 0.161, *η*^2^ = 0.02), the level of BA in men differed significantly for the different BMI-groups, Welch’s *F*(3, 266.36) = 5.06, *p* = 0.007, *η*^2^ = 0.04. Games–Howell post-hoc analysis revealed a significant difference between BAS scores of the groups with obesity and normal-weight (*p* = 0.005; − 0.24, 95% CI [− 0.43, − 0.06]). No significant differences were found between the overweight group and the normal-weight group (*p* = 0.250; − 0.09, 95% CI [− 0.22, 0.04]), and between the overweight group and the obese group (*p* = 0.110; − 0.16, 95% CI [− 0.34, 0.03]).

The level of AE differed statistically significantly for the BMI-groups in men (Welch’s *F*(2, 273.19) = 16.37, *p* < 0.001, *η*^2^ = 0.11) and women (Welch’s *F*(2, 184.53) = 16.89, *p* < 0.001, *η*^2^ = 0.15).

In men, Games–Howell post-hoc analysis revealed participants in the obese group to report significantly lower AE compared to the normal-weight (*p* < 0.001; − 0.47, 95% CI [− 0.66, − 0.27]), and overweight group (*p* < 0.001; − 0.37, 95% CI [− 0.57, − 0.18]). The overweight group did not significantly differ from the normal-weight group (*p* = 0.324; − 0.09, 95% CI [− 0.25, 0.06]).

In women, the obese group reported significantly lower AE compared to the normal-weight (*p* < 0.001; − 0.49, 95% CI [− 0.72, − 0.27]), but not the overweight group (*p* = 0.058; − 0.24, 95% CI [− 0.49, 0.01]). The overweight group reported lower AE compared to the normal-weight group (*p* = 0.001; − 0.25, 95% CI [− 0.41, − 0.09]).

Participants’ BMI, gender, age, education, depressive symptoms and self-esteem explained a significant proportion of variance in BA (*R*^2^ = 0.29, *F*(9, 907) = 42.00, *p* < 0.001) and AE scores (*R*^2^ = 0.31, *F*(9, 903) = 47.40, *p* < 0.001).

A significant interaction between BMI and depressive symptoms (*p* < 0.001) as well as BMI and self-esteem (*p* < 0.001) with regard to BA was found. In contrast to individuals with obesity (*r* = − 0.352, *p* < 0.001), overweight (*r* = − 0.377, *p* < 0.001), and normal-weight (*r* = − 0.403, *p* < 0.001), the association between BA and depressive symptoms was not statistically significant in individuals with underweight (*r* = − 0.220, *p* = 0.376). The relation between BA and self-esteem was highest in the obese group (*r* = 0.658, *p* < 0.001) and decreased from the overweight (*r* = 0.373, *p* < 0.001) to the normal-weight group (*r* = 0.282, *p* < 0.001), while no statistically significant link was observed in the underweight group (*r* = − 0.129, *p* = 0.623).

Further, a significant interaction effect was found between BMI and depressive symptoms regarding AE (*p* = 0.002). There was a statistically significant relationship observed between the two variables in individuals with underweight (*r* = − 0.341, *p* = 0.162) in contrast to individuals with obesity (*r* = − 0.305, *p* < 0.001), overweight (*r* = − 0.327, *p* < 0.001) and normal-weight (*r* = − 0.385, *p* < 0.001).

All variables and their statistic contribution to the prediction of the body image scores are illustrated in Tables 2 and 3.

**Table 2:** Multiple linear regression analysis using BA as criterion

	*B*	SE	*β*	*T*	*p*	95% CI
Constant	4.049	0.082		49.581	< 0.001	3.888, 4.209
BMI	− 0.019	0.003	− 0.164	− 5.535	< 0.001	− 0.025, − 0.012
Gender	− 0.062	0.035	− 0.051	− 1.779	0.076	− 0.130, 0.006
Age	0.004	0.001	0.113	3.738	< 0.001	0.002, 0.006
Education	− 0.003	0.021	− 0.005	− 0.157	0.875	− 0.045, 0.038
Depressive symptoms	− 0.031	0.004	− 0.238	− 7.920	< 0.001	− 0.038, − 0.023
Self-esteem	0.204	0.020	0.311	10.435	< 0.001	0.166, 0.243
BMI × age	0.000	0.000	0.016	0.544	0.586	0.000, 0.000
BMI × depression	0.002	0.001	0.143	4.130	< 0.001	0.001, 0.004
BMI × self-esteem	0.024	0.004	0.211	6.113	< 0.001	0.017, 0.032

Model fit calculated from valid cases: *F*(9, 907) = 42.00, *p* < 0.001, adjusted *R*^2^ = 0.287

*B* unstandardized regression coefficient, *SE* standard error, *β* standardized regression coefficient, *T* *t* test, 95% *CI* confidence interval

**Table 3:** Multiple linear regression analysis using AE as criterion

	*B*	SE	*β*	*T*	*p*	95% CI
Constant	4.450	0.106		40.017	< 0.001	3.447, 3.802
BMI	− 0.168	0.029	− 0.114	− 9.839	< 0.001	− 0.044, − 0.030
Gender	0.000	0.001	− 0.009	0.946	0.345	− 0.039, 0.112
Age	− 0.035	0.003	− 0.229	1.248	0.212	− 0.001, 0.004
Education	− 0.059	0.013	− 0.101	0.686	0.493	− 0.030, 0.063
Depressive symptoms	0.004	0.014	0.006	− 6.280	< 0.001	− 0.035, − 0.019
Self-esteem	− 0.115	0.014	− 0.181	13.002	< 0.001	0.240, 0.325
BMI × age	0.020	0.014	0.034	1.767	0.078	0.000, 0.001
BMI × depression	0.041	0.017	0.066	3.045	0.002	0.001, 0.003
BMI × self-esteem	0.063	0.019	0.079	0.189	0.851	− 0.008, 0.010

Model fit calculated from valid cases: (AE) *F*(9, 903) = 47.40, *p* < 0.001, adjusted *R*^2^ = 0.314

*B* unstandardized regression coefficient, *SE* standard error, *β* standardized regression coefficient, *T* *t* test, 95% *CI* confidence interval

## Discussion

The aim of the current study was to explore BA in a representative German population sample and to compare BA between men and women as well as different BMI groups. Further, the relationship of the positive (BA) and negative (AE) body image facet with the variables BMI, gender, age, education, depressive symptoms and self-esteem was analyzed.

Contrary to our hypothesis (a), women and men did not differ significantly in their BA scores. Previous studies with German samples have found both, women to report lower BA levels [25], and higher BA levels compared to men [33]. In addition, the current result is not unprecedented: Men and women did not differ significantly in BA in a study by Razmus [24], and Tylka and Wood-Barcalow [23] also report no differences in BA means between men and women in their community sample. Moreover, age was identified as a significant contributor to the prediction of BA scores in the current sample. This finding might also serve as an explanation for the non-significant gender difference in BA in the current sample: On average, male participants were significantly younger than female participants. Previous studies in women report BA levels to increase across age, with older women reporting higher BA [34], while BA levels in men have been found to remain stable over different ages [33].

Moreover, hypothesis (b) could be confirmed in male participants, but not in female participants. Significantly lower BA scores were observed in male participants with obesity compared to participants with normal-weight, while in women the BMI-groups did not differ significantly in BA. The current results join a line of inconsistent findings regarding the relationship between BMI and BA: In previous studies, the association between the two variables was either not significant [26] or negative, with participants with obesity reporting lower BA compared to normal-weight participants [18, 24]. Contrasting the current results, Swami and colleagues [25] report the negative association between BA and BMI for women but not for men. Interestingly, BA scores in the current sample reached rather positive values (*M*_total_ = 3.94) even in participants with obesity (*M*_male_ = 3.80; *M*_female_ = 3.79) and the significant differences in men could only be found between the aforementioned two out of the four BMI-groups. Thus, it cannot be ruled out that this result is an incidental finding that might be attributed to the sample size and thus statistical power of the current study.

Further, with the exception of individuals with underweight, lower levels of depressive symptoms were significantly associated with higher levels of BA in individuals with normal-weight, overweight, and obesity. This is in line with previous studies reporting a negative association between BA and depressive symptoms [35, 36]. With regard to the results in individuals with underweight, the small sample size compared to the other BMI groups needs to be taken into consideration: Judging from the correlation coefficient, the link between BA and depressive symptoms takes the same direction in the underweight group as it does in the other BMI groups and thus might not have reached significance due to a lack of statistical power rather than specific theoretical reasons. Similarly, higher self-esteem was significantly associated with higher levels of BA in all BMI groups except the underweight group. Swami and colleagues [25] report similar findings in their study: BA was the only significant predictor of self-esteem in both, men and women. Also, Gillen [37] observed individuals with greater positive body image to report higher self-esteem even after controlling for BMI.

With regard to the negative body image facet, hypothesis (a) was not confirmed, as men reported significantly lower AE scores compared to women in the current sample. While previous research has often found women to be more dissatisfied with their body compared to men [38, 39], there are also studies reporting no differences between men and women in AE [40]. Previous research has pointed out that in recent times, body dissatisfaction in men has been reaching similar prevalence levels to body dissatisfaction in women [41, 42]. Taken together with the inverse relationship between body dissatisfaction and BMI, it does not seem unreasonable that men in the current sample—given that their BMI was higher compared to female participants—reported lower AE levels. In addition, men and women reported on average AE scores slightly above the value of 3.5, which corresponds to a marginally positive evaluation of their appearance. Male participants therefore might have been more neutral in their evaluation compared to female participants, rather than more negative. Unlike in BA, age did not significantly contribute to the prediction of AE scores in the current sample. Previous research has also found stable body dissatisfaction levels across different ages in both, men and women [33].

The current results confirmed hypothesis (b), and were in line with previous studies regarding differences in AE scores between BMI groups [12]: A significant inverse relationship between BMI group and AE was found in both, male and female participants.

Finally, with the exception of individuals with underweight, lower levels of depressive symptoms were significantly associated with higher levels of AE in individuals with normal-weight, overweight and obesity. These results correspond to previous research that has found positive associations between body dissatisfaction and depression in men [43] and women [44].

The present results are particularly interesting in relation to body image concerns in obesity since they illustrate the importance of examining both, positive and negative body image, and the relationship between these two independent constructs in this population. While AE in individuals with obesity was lower compared to normal-weight individuals in men and women, thus reinforcing previous findings, no such consistent difference could be established for BA. Since obesity (and its treatment) is primarily associated with negative consequences for, and limitations of the body, individuals with obesity are frequently confronted with their bodily deficits. Exploring BA as either protective factor against body image threats or starting point for interventions addressing body image concerns, might be a promising way to make individuals with obesity aware of their bodily resources as well.

The following limitations should be taken into consideration when interpreting the current results: Due to the cross-sectional nature of the study, the associations between the analyzed outcome variables cannot be interpreted as causal in nature. Potential reciprocity of the relationship between the outcome variables, especially with regard to BA, AE, depression and self-esteem ultimately needs to be determined using longitudinal data. Moreover, since body image is primarily influenced by our social experiences and since sociocultural influences are subject to change over time [8] longitudinal studies that also include different birth cohorts could draw a more detailed picture of positive and negative body image in the German population and also investigate the growing influence of social media in the development of body image in general and body image concerns in particular [45].

Further, BMI was calculated from self-reported height and weight data. Previous studies have found underestimation of BMI derived from self-reported height and weight especially in individuals with overweight and obesity [46]. While it cannot be ruled out that participants underestimated their weight and overestimated their height, BMI values in the current study are comparable to BMI values determined by anthropometric measurements in a representative sample of the adult German population [47].

Moreover, due to its exploratory nature and time constrains regarding the telephone survey, measurement of body image in its full multidimensionality was unfeasible in the current study, and assessment was limited to one positive (BA) and one negative (AE) body image facet. To broaden our knowledge on positive body image as well as its relationship with negative body image, future studies on the subject should investigate other body image dimensions, like for example body image perception, body image flexibility or functional body image. The latter might be of particular interest in overweight and obesity considering their detrimental effects on various body functions like movement due to for example musculoskeletal disorders, osteoarthritis, and respiratory disease [48–50]. Also due to time and budget constraints with regard to the telephone survey, assessment of self-esteem was constrained to the use of a single item rather than the use of a more comprehensive instrument. While recent studies have found single-item scales valid and reliable in assessing global self-esteem [51], the current results regarding self-esteem nevertheless should be understood as exploratory and interpreted with the necessary caution. Consequently, complete assessment of body image as well as self-esteem could be a valuable endeavour for future studies on the subject.

Finally, the current study analysed data of individuals of the general public. However, when it comes to body image concerns, particularly individuals with obesity are not a homogenous group. Previous studies have identified individuals seeking obesity treatment like bariatric surgery as well as individuals with obesity suffering from binge eating disorder to be particularly affected by negative body image [15, 52]. Future research investigating positive body image in particularly vulnerable subgroups with obesity in addition to its relationship with frequently identified outcome variables in the context of body image like self-esteem and depression might be important to improve current or develop new treatment options.

In conclusion, the present study is one of few studies so far that investigated positive body image in a representative population sample in Germany. Ten years ago, a nation-wide survey in the general German population found concern about at least one body part in 35.3% of participants [53], highlighting the relevance of negative body image even in the “average citizen”. Broadening the view on body image by including positive body image facets and studying their prevalence and specific manifestation in the general public could help identify protective factors or populations at risk, and help estimate the demand for treatment programs. While previous studies focused on negative body image facets like body dissatisfaction, and body image in young women, in the current study BA was analyzed in individuals of different BMI groups as well as in men and women to gain a more complete picture. BA reached rather high values and did not differ between male and female participants. Also—with the exception of male participants with obesity and normal-weight—no differences were found between BMI groups, reinforcing preliminary evidence [54] that BA could potentially serve as protective factor against body image-related threats. Considering how individuals with obesity in particular are frequently confronted with their “failure” to comply with societal body ideals, identifying resources rather than deficits to aid coping or even treatment of their body image concerns, is crucial.

## What is already known on this subject?

Negative body image is prevalent in obesity, and linked to e.g. psychological distress. Previous studies on positive body image in individuals with obesity are rare, and results inconsistent.

## What your study adds?

Participants with obesity reported lower body appreciation compared to normal-weight participants, but only in men. No difference in BA scores was found between male and female participants.

## Data Availability

The datasets generated and analyzed during the study are currently not publicly available but are available from the corresponding author on reasonable request.
